# The first full host plant dataset of Curculionidae Scolytinae of the world: tribe Xyleborini LeConte, 1876

**DOI:** 10.1038/s41597-023-02083-5

**Published:** 2023-03-25

**Authors:** Enrico Ruzzier, Giacomo Ortis, Davide Vallotto, Massimo Faccoli, Isabel Martinez-Sañudo, Matteo Marchioro

**Affiliations:** Department of Agronomy, Food, Natural Resources, Animals and Environment (DAFNAE), Università degli Studi di Padova, Legnaro, 35020 Italy

**Keywords:** Entomology, Forestry

## Abstract

Xyleborini is the largest tribe of Scolytinae accounting for about 1300 species worldwide; all species are primarily xylomycetophagous, developing on symbiotic fungi farmed in plant woody tissues. Xyleborini wood-boring action, associated with the inoculum of symbiotic fungi, can lead, sometimes, to the emergence of host plant dieback, wood damage and death; for this reason, multiple Xyleborini are major pests on both cultivated, forest and ornamental trees. Many Xyleborini are invasive worldwide and great effort is expended to manage their biological invasions or prevent new arrivals. Imports of host plants often have a primary role as a pathway for introduction and are frequently responsible for the establishment of species in non-native environments. In this context, data availability on Xyleborini host plants is a major limiting factor in the development of effective detection and monitoring strategies as well as a fundamental variable to consider in risk assessment of plant pests and invasive species. This contribution provides updated host records and the hosts economic categorization for the 1293 Xyleborini known worldwide to date.

## Background & Summary

The Scolytinae (Coleoptera: Curculionidae) is a highly diverse subfamily of weevil beetles inhabiting all regions of the world, except Antarctica^1^. Following present interpretations, Scolytinae is a polyphyletic assemblage of weevils united by their adaptation to bore in plant tissues at the adult stage^2,3^. This group apparently evolved during the mid-Cretaceous in parallel with the synchronous differentiation of Spermatophyta^4^. Living species are associated with almost all groups of terrestrial herbaceous and arboreous plants and are adapted to feed and develop on almost all plant parts^5^. These adaptations corresponded with the evolution of multiple morphological, ecological and ethological traits such as the development of compact, cylindrical bodies with a short rostrum that provides powerful mandibles that could easily bore into plant tissues, the capability to feed on plant parts other than xylem, and the evolution of mutualistic relationships with microorganisms^6^. Due to their evolutionary plasticity and adaptability, Scolytinae are capable of developing on healthy, dead, or dying plants, consequently playing a unique ecological role in forest ecosystems (*e.g*.^7,8^).

Xyleborini LeConte, 1876 is the largest tribe in Scolytinae, with almost 1300 described species distributed into 43 genera^9–17^. The tribe inhabits all major world climatic regions and its highest diversity can be found in tropical and subtropical regions. Xyleborini are generally xylomycetophagous species^18^, rarely seed feeders^19–21^, and actively farm symbiotic fungi inside parental galleries^22,23^. These fungi serve primarily as food for both larvae and adults but also to detoxify plant defense chemicals^24^. The infection caused by the fungi, in association with the damage caused by the boring activity of the beetles, usually causes the onset of a series defensive responses by the affected plant, such as sap oozing and gumming. In some cases, the infection caused by the fungus can cause the dieback or death of the host plant (*e.g*.^25,26^).

Many xyleborine species are significant for agriculture and forestry because they are pests^27–31^. In addition, Xyleborini include many non-native invasive species introduced worldwide^32–38^. As is the case for many other alien species, their interactions with both non-native and indigenous plants may cause socio-economic (yield loss, trade restriction, or change in market values) or environmental impacts (disturbance of natural ecosystem processes, influences on populations of native species)^39,40^.

Consequently, considerable effort has been made worldwide to understand and predict the main pattern of non-native species arrival^41–43^ and, where possible, to prevent species introduction through the development of effective survey and detection methodologies and tools^44–48^. In this perspective, information on host plants and host range are two of the key factors explaining species dispersal, introduction pathways, and establishment success^41^. Furthermore, host preference and host diversity might have a relevant role in Xyleborini management^49^.

To date, despite a growing interest in host plants for phytosanitary or analytical purposes, much of the data on host associations remains scattered through the extensive literature regarding Scolytinae, with only a few exceptions such as monographs and catalogs, e.g.^50^. To meet this need we intend to start a series of contributions specifically aimed at summarizing and updating the knowledge about the host species of not only Xyleborini but all Curculionidae Scolytinae known worldwide, providing a new complete and updated resource for future research.

## Methods

### Host plant definition

In compiling these data, we identified host plant records based on all records of xyleborine species observed boring inside any plant part or tissue. Records derived from trapping or other observations of occurrence in plantations (including in monocultures) were considered unreliable and therefore not included in the dataset.

### Data collection

The creation of the host dataset started from drafting of the most updated species list of Xyleborini in the world. This checklist was initially based on the Wood & Bright catalog^50^ and following supplements^50–54^, and it was subsequently integrated on the basis of all taxonomic papers on Xyleborini published afterwards. The current version of the database includes all Xyleborini species known and described prior to October 30th 2022.

The research of host plants was conducted through a systematic search, treating individual Xyleborini species one at a time. The search for host plants was performed by searching not only for the beetle’s valid name, but also by using its synonyms. The research was performed in Google Scholar and Google for each species on the list through the use of selected keywords such as the species name of the scolytine (*e.g*., “*Xylosandrus amputatus*”) also in combination with other keywords such as “host”, “pest” (integrated by the usage of the Boolean operators “AND”, “OR”, “NOT” and the use of double inverted commas for specific word combinations). Furthermore, data collection was integrated through the extensive revision of reports (annual, research, technical, project, etc.), working papers, government documents, evaluations and a series of websites and online resources^55,56^, as well as books, catalogs and manuals. The sources consulted were not limited to those in English, but included other languages (e.g. French, German, Italian, Portuguese, Spanish) and idioms (e.g. Chinese, Japanese, Russian) using, where necessary, instant translation sites (DeepL or Google translate). In order to guarantee a search as precise and exhaustive as possible, a reiterated host research was made for each xyleborine species until the results no longer produced new records.

Plant taxonomy adopted in the dataset was based on the information available from the “Plant of The World Online” database (POWO)^57^, whereas the economic uses of plant species were based on the “U.S. National Plant Germoplasm System” database^58^. Economic use of plants was organized in three categories: 1) Agricultural (plants used in medicine -also folklore-, human and animal food, agroforestry, materials -beads, gum/resin, essential oils, poison, tannin/dyestuff, etc.); 2) Forest (plants used as wood, fuel, furniture); 3) Ornamental (plants with ornamental, shade and shelter, domestic usage).

The references included in the database were both the most relevant, the most updated and those specifically referring to a determined xyleborine species and its hosts. In case of multiple references referring to the same species or reporting the same information, only one, and generally the first recovered or the most exhaustive, was selected and included in the database. Uncertain or imprecise records, including records using vernacular or local names, that could not be traced back to a reliable host species, were not included in the dataset.

## Data Records

The database for the host plants of Scolytinae Xyleborini of the world is available on Zenodo with the original database in XLSX format (i.e. “Complete_dataset_Xyleborini.xlsx”)^59^; the reference list is included in the same file as a different spreadsheet named “References”. The database is organized in eight columns as follows: “Species”, where the full name of the xyleborine species are provided in association with the describer’s name; “Host Family”; “Host Genus”; “Host Species”; “Reference”, and the three economic categories “Agricultural”, “Forest” and “Ornamental”. In the dataset, xyleborine species are sorted alphabetically, both by genus and by species. Plant family and genus records do not imply that a specific xyleborine species feeds on all the plants belonging to that category, but instead that this data is the most specific/detailed information available in the reviewed literature suggesting that a determined xyleborine species feeds on at least one plant species belonging to that specific family/genus.

The database will be periodically updated with new versions (named Version 1.0 onwards); the latest and most updated database will be the first to access via the DOI provided here, however previous versions of the same file will also remain available in the repository. The first version (1.0) provides information for 1293 species of Xyleborini, of which 535 have no host records available. For the 758 species in which the host is known, 574 species have their host known at species level while for 184 the host is limited to family or genus. The dataset includes records for 2188 plant species, distributed among 178 families and 1027 genera; 723 plants belong at least to one of the three economic categories considered.

## Technical Validation

All host records included in the database are based on articles published in scientific journals, books, reports, and databases managed by the major leading experts on scolytine beetles (e.g. Atkinson database: Bark and Ambrosia Beetles of the Americas) and international phytosanitary agencies (e.g. EPPO^60^, CABI^61^); therefore, we have confidence in their accuracy, frequently guaranteed by the peer-review process.

As already specified in materials and methods, to standardize and harmonize the information we critically reviewed all the data collected, keeping only those relating to xyleborine species whose relationship with the host plant could be recognized unequivocally; for this reason, we have excluded all possible cases that do not fall within the standards defined in the materials and methods section. Each record in the dataset is associated with the respective bibliographic source, allowing users to both assess the validity of the record and interpret and reuse the data. We listed the references cited in the database, making it possible for users to access the original sources.

Xyleborini taxonomy is standardized following the International Code of Nomenclature Zoology (ICZN)^62^. The complete list of genera and species belonging to the Xyleborini family was compiled using the Bright, 2021 catalog^54^, integrated with the latest publications and, finally, cross-validated by Sarah M. Smith and Anthony I. Cognato (Michigan State University), two of the world’s leading experts on Xyleborini. Plant taxonomy follows the International Code of Nomenclature for algae, fungi, and plants^63^, and taxa names and authors, including those of subspecies, varieties and hybrids are consistent with those provided in the internationally recognized Plants of the World Online database and the International Plant Names Index (IPNI)^64^.

Our aim is to keep updating the xyleborine species list and host plant data in the future. Data will be corrected and updated if any errors or updates are reported to the first or last author (enrico.ruzzier@unipd.it or matteo.marchioro@unipd.it).

## Usage Notes

We would appreciate if researchers cite the database stored in Zenodo^59^ in the specific version used, as well as this publication, when using all or part of the database.

## Data Availability

No custom code was used to generate or process the data described in the manuscript.
